# The structure and demographic correlates of cancer fear

**DOI:** 10.1186/1471-2407-14-597

**Published:** 2014-08-16

**Authors:** Charlotte Vrinten, Cornelia H M van Jaarsveld, Jo Waller, Christian von Wagner, Jane Wardle

**Affiliations:** Department of Epidemiology and Public Health, Cancer Research UK Health Behaviour Research Centre, UCL, Gower Street, London, WC1E 6BT UK; Department of Primary Care and Public Health Sciences, King’s College London, Capital House, 42 Weston Street, London, SE1 3QD UK

**Keywords:** Cancer fear, Cancer worry, Anxiety, Education, Ethnicity, Gender, STAI, Older adults

## Abstract

**Background:**

Cancer is often described as the ‘number one’ health fear, but little is known about whether this affects quality of life by translating into high levels of worry or distress in everyday life, or which population groups are most affected. This study examined the prevalence of three components of cancer fear in a large community sample in the UK and explored associations with demographic characteristics.

**Methods:**

Questions on cancer fear were included in a survey mailed to a community sample of adults (n = 13,351; 55–64 years). Three items from a standard measure of cancer fear assessed: i) whether cancer was feared more than other diseases, ii) whether thinking about cancer caused discomfort, and iii) whether cancer worry was experienced frequently. Gender, marital status, education, and ethnicity were assessed with simple questions. Anxiety was assessed with the brief STAI and a standard measure of self-rated health was included.

**Results:**

Questionnaire return rate was 60% (7,971/13,351). The majority of respondents agreed or strongly agreed that they feared cancer more than other diseases (59%), and felt uncomfortable thinking about it (52%), and a quarter (25%) worried a lot about cancer. All items were significantly inter-correlated (r = .35 to .42, p’s < .001), and correlated with general anxiety (r = .16 to .28, p’s < .001) and self-rated health (r = -.07 to -.16, p’s < .001). In multivariable analyses including anxiety and general health, all cancer fear indicators were significantly higher in women (ORs between 1.15 and 1.48), respondents with lower education (ORs between 1.40 and 1.66), and those with higher general anxiety (ORs between 1.50 and 2.11). Ethnic minority respondents (n = 285; 4.4%) reported more worry (OR: 1.85).

**Conclusions:**

More than half of this older adult sample in the UK had cancer as greatest health fear and this was associated with feeling uncomfortable thinking about it and worrying more about it. Women and respondents with less education or from ethnic minority backgrounds were disproportionately affected by cancer fear. General anxiety and poor health were associated with cancer fear but did not explain the demographic differences.

## Background

Cancer occupies an almost unique position among diseases in terms of the fear it engenders. The word ‘cancer’ was once considered unacceptable in the public sphere, and even today, euphemisms such as ‘the Big C’ are common. In the 1950s, the British Empire Cancer Campaign concluded that education about early symptoms of cancer in Britain would create mass panic [1]; and similar issues have been raised in connection with campaigns to promote self-examination for early signs of testicular cancer in the UK [2]. Polls in the US and Europe find that at least half the population say they fear cancer more than any other disease [3–5], and around a third to a fifth say they fear cancer more than other potential catastrophes, such as violent crime, debt, and losing a job [3, 6].

Fear is an unpleasant emotion and the pervasiveness of cancer fear in the population may have implications for quality of life. In addition, cancer fear has been shown to be associated with screening uptake and presentation of suspicious symptoms, although both motivating and deterrent associations have been found (for an overview, see [7, 8]). In the light of the frequency of public statements about cancer fear, it is clearly a societally important matter. Most research to date has examined the behavioural consequences [7–10], and studies that have focused on cancer fear itself are mostly qualitative (e.g. [11]) or done in undergraduate student samples for whom the threat of cancer is less relevant due to their young age [12, 13]. A better understanding of the nature of cancer fear may help identify those who suffer from maladaptive and undue fears, and help explain why the behavioural responses seem to vary.

Fear as an emotion has a complex architecture, with cognitive, physiological and affective components that may be only loosely interconnected. These components are often not distinguished in the cancer context, and terms such as ‘cancer fear’ and ‘cancer worry’ are sometimes used synonymously (for example, see [7, 8, 14]). A failure to distinguish between different fear components may have contributed to the apparent inconsistencies in the behavioural effects of fear. For example, appraising cancer as uniquely frightening may lead to avoidance of the fear stimulus, while worry may encourage behaviours that will result in reassurance. Appraising cancer as uniquely frightening cannot therefore be assumed to translate into high levels of worry or avoidance [13] or show the same behavioural outcomes. To date, no studies have examined the associations between the appraisal of cancer as frightening, discomfort when thinking about cancer, and worry about cancer, nor investigated whether demographic and psychosocial correlates are consistent across the different components of cancer fear. Large datasets that include different indicators of cancer fear are needed to examine the interconnections between different fear components.

Some previous studies have explored associations between different aspects of fear, although these were mainly conducted in the context of specific cancer screening programmes. Consedine et al. [15] explored three aspects of fear: trait anxiety, prostate cancer worry, and screening fear, in a sample of older men in the US. All three were associated with lower income and education, and prostate cancer worry and screening fear, but not trait anxiety, was higher in Black men. Another study from the US found strong associations between cancer worry and cancer-related discomfort among women with and without a family history of breast cancer, but only moderate associations between cancer worry and trait anxiety [13]. Another examined the contributions of cancer worry and cancer-related distress to breast screening uptake in women at an increased familial risk, but did not report the associations between the different fear components [16]. No large studies on inter-relationships between the components of cancer fear have been conducted outside the US.

Little is known about the demographic correlates of individual fear components. Polls in the US and Europe that show cancer to be uniquely frightening have not explored whether certain subgroups are more likely to endorse this view, although a French survey noted that more women than men viewed cancer as their ‘number one’ fear [4]. The 2003 Health Information National Trends Survey (HINTS; [17]) and the Pittsburgh Lung Screening study [18] both showed higher cancer worry in women. Lower socioeconomic status (SES) was associated with cancer worry in both these studies, and in the UK Flexible Sigmoidoscopy Trial [19]. Ethnic minority status has been linked with higher cancer worry in studies in the US and UK [15, 20–22], although the association has differed by type of cancer and specific ethnic background [17, 23]. The reason that so little is known about the correlates of general cancer fear is partly that much previous work measured single components of cancer fear and focussed specifically on associations with screening uptake, without exploring the population distribution of fear (e.g. [15, 16, 21]).

An important potential confounder in studies of demographic variation in cancer fear is general anxiety. Anxiety tends to be higher among women and more socially disadvantaged groups [24, 25], so might explain sex or education differences in cancer fear. Results have been more varied in relation to ethnicity. African American men showed lower trait anxiety than White Americans despite higher prostate cancer worry in one study [15]. In the HINTS results, controlling for psychological distress reduced both gender and ethnic differences in cancer worry, although multiple other behavioural factors were also included as control variables, making it difficult to identify whether psychological distress was the key confounder. High trait anxiety has also been shown to increase the effect of media breast cancer messages on breast cancer fear [26].

The present study aimed to examine associations between three indicators of cancer fear that represent different components (having cancer as greatest health fear, discomfort thinking about cancer, and cancer worry) and associations between all three and general anxiety. It also explored the demographic correlates of the three components and examined whether effects were explained by differences in general anxiety and self-rated health. There is no prima facie reason to believe that the architecture of cancer fear would be different across cultures, but the socio-demographic correlates may vary between countries because of differences in healthcare provision, public knowledge of cancer, or beliefs about cancer prevention. Few previous studies of cancer fear have been conducted in the UK, a country that has a well-organised health care system, but also a tradition of the ‘stiff upper lip’, and a history of reluctance among health professionals to provide much public information about cancer for fear of scaring the public.

## Methods

### Design and procedure

Data for this secondary data analysis come from the baseline questionnaires mailed between 1996 and 1999 to all adults aged 55–64 years (i.e. born between 1932 and 1943) registered in 506 General Practices taking part in the UK Flexible Sigmoidoscopy (FS) Trial. This was a multi-centre, randomised controlled trial to evaluate the efficacy of FS screening on colorectal cancer incidence and mortality [27, 28]. Cancer worry, general anxiety, and attitudes and beliefs about cancer and screening were also assessed in a subset of Practices [27]. Potential participants were identified by Health Authorities, and GPs were asked to exclude any patients who were ineligible (a history of colorectal cancer, adenomas or inflammatory bowel disease, severe disease or a life expectancy of less than five years, endoscopic colorectal examination within the past three years). This excluded 7,602 participants (2%; [29]). The remaining participants (n = 368,142) were sent an information letter about the study together with the baseline questionnaire. In a subsample of Practices, with a total of 13,351 eligible adults, the baseline questionnaire included questions on cancer fear, as well as a range of demographic, health, and psychosocial measures. The UK FS Trial was conducted in accordance with the Declaration of Helsinki and approval was obtained from the local research ethics committee for all participating centres.

### Measures

Cancer fear was assessed with three items adapted from Berrenberg’s Cancer Attitude Inventory [30]: i) ‘Of all the diseases there are, I am most afraid of cancer’ (‘cancer as greatest health fear’), ii) ‘It makes me uncomfortable to think about cancer’ (‘discomfort thinking of cancer’), and iii) ‘I worry a lot about cancer’ (‘cancer worry’). The Cancer Attitude Inventory is a 41-item measure of attitudes towards cancer that encompasses a range of domains including cancer stigma, economic hardship, and potential for positive growth. The three items used in this study were chosen as potentially representing different aspects of cancer fear. All items used a 5-point Likert response scale from ‘strongly disagree’ to ‘strongly agree’. For the chi-square analyses and the multivariable logistic regression analyses, responses of ‘agree’ or ‘strongly agree’ were combined to define the higher fear response (i.e. those who agreed with the fear statement).

General anxiety was assessed with the 6-item State version of the Spielberger State Trait Anxiety Inventory [31]. Total scores ranged from 6 to 24, and were dichotomised for the chi-square and multivariable logistic regression analyses. For ease of interpretation of the results, groups scoring below or above the group average (<11 vs. ≥11) were created. Self-rated general health was included as a control variable and assessed with the question: ‘Would you say that for someone of your age your own health in general is’: ‘poor’, ‘fair’, ‘good’, ‘excellent’ [32]. For binary analyses responses were dichotomised into ‘poor or fair’ and ‘good or excellent’.

Demographic data came either from the GP database (age and sex) or were assessed in the questionnaire (ethnicity, marital status, education). Age was dichotomised into ‘younger than 60’ and ‘60 years or older’, to aid interpretation of the results. Ethnicity was reported using 5 categories (‘White’, ‘Black’, ‘Asian’, ‘other’, and ‘prefer not to say’), but for these analyses, ‘Black’ (n = 79), ‘Asian’ (n = 166) and ‘other’ (n = 40) were combined because the numbers in each individual group were small, and ‘prefer not to say’ was coded as missing. Marital status was reported in 5 categories (‘married/living as married’, ‘divorced’, ‘separated’, ‘widowed’, ‘single’), and dichotomised into ‘married or cohabiting’ and ‘not married or cohabiting’. Education was assessed with a single item (‘do you have any educational qualifications, e.g. School Certificate, GCE O’Levels, etc.’) with a ‘yes’ and ‘no’ answer. These are examinations taken at age 16 in the UK. In the cohort born between 1932 and 1943 in the UK, continuation in education would have been dependent on passing these examinations. Education has been shown to be a good measure of SES in older adults [33].

### Statistical analysis

To examine associations between the three fear indicators, general anxiety, and general health, we calculated Spearman’s correlations using the values before dichotomisation. To explore whether having cancer as the greatest health fear translated into high levels of worry or discomfort, we explored associations between the three fear indicators using chi-square tests for the dichotomised items. Univariate chi-square analyses were then used to examine demographic correlates of each cancer fear indicator using dichotomised values. To explore the consistency in the demographic correlates of the three fear components while controlling for differences in general anxiety and self-rated general health, two sets of multivariable logistic regression analyses using the dichotomised items were carried out: one that only included the demographic variables as predictors of each separate fear indicator (Model 1), and one that controlled for anxiety and self-rated health (Model 2). Because of the multiple comparisons, a Bonferroni correction was applied to control the family-wise error rate for an overall alpha level of .05, and thus a p-value of .001 was used to indicate statistical significance. The sample for analysis consisted of respondents with complete information on all study variables. SPSS version 20.0 was used for all analyses.

## Results

The questionnaire was mailed to 13,351 adults in the eligible age range in participating General Practices. The return rate was 59.7% (n = 7,971), of which 6,527 (82% of responses) had complete data on all variables. There were slightly more women (53%) than men (47%). More than half the respondents had no educational qualifications (63%), and the majority were of White ethnic origin (96%) and married or cohabiting (75%). The mean STAI score was 10.6, which is comparable to other community-based studies of older adults [34, 35]. Most respondents (70%) rated their health as good or excellent.

Over half the respondents agreed (or strongly agreed) that: ‘Of all the diseases there are, I am most afraid of cancer’ (59%), and almost as many (52%) agreed that: ‘It makes me uncomfortable to think about cancer’. A smaller proportion (25%) agreed with: ‘I worry a lot about cancer’. Characteristics of the sample are presented in Table 1.

**Table 1:** Associations with demographic factors, health status, and anxiety for each cancer fear indicator*

Characteristic (n)	Whole sample	Cancer as greatest health fear		Discomfort thinking about cancer		Cancer worry	
	%	%	Significance	%	Significance	%	Significance
Whole sample (6,527)	100	58.7	-	52.3	-	24.9	-
Gender							
Male (3,043)	46.6	54.7	*χ* ^2^ = 37.88	49.6	*χ* ^2^ = 16.84	20.4	*χ* ^2^ = 60.66
Female (3,484)	53.4	62.2	p < .001	54.7	p < .001	28.8	p < .001
Age							
< 60 years (3,300)	50.6	59.2	*χ* ^2^ = 0.83	52.8	*χ* ^2^ = 0.48	24.8	*χ* ^2^ = 0.001
≥ 60 years (3,227)	49.4	58.1	p = .36	51.9	p = .49	24.9	p = .97
Educational qualifications							
Yes (2,412)	37.0	50.7	*χ* ^2^ = 100.99	46.1	*χ* ^2^ = 58.81	20.2	*χ* ^2^ = 43.97
No (4,115)	63.0	63.4	p < .001	56.0	p < .001	27.6	p < .001
Ethnicity							
White (6,242)	95.6	58.5	*χ* ^2^ = 1.46	52.1	*χ* ^2^ = 3.24	24.3	*χ* ^2^ = 24.24
Not White (285)	4.4	62.1	p = .23	57.5	p = .07	37.2	p < .001
Marital status							
Married or cohabiting (4,877)	74.7	58.7	*χ* ^2^ = 0.01	51.5	*χ* ^2^ = 5.32	23.8	*χ* ^2^ = 10.73
Not married or cohabiting (1,650)	25.3	58.5	p = .91	54.8	p < .05	27.9	p < .01
General health							
Excellent/good (4,591)	70.3	57.3	*χ* ^2^ = 11.37	49.6	*χ* ^2^ = 46.56	21.3	*χ* ^2^ = 102.64
Fair/poor (1,936)	29.7	61.8	p < .01	58.8	p < .001	33.2	p < .001
Anxiety							
Low (3,624)	55.5	53.8	*χ* ^2^ = 81.05	45.0	*χ* ^2^ = 176.87	17.4	*χ* ^2^ = 244.14
High (2,903)	44.4	64.8	p < .001	61.5	p < .001	34.2	p < .001

*Percentages for the cancer fear indicators represent those with higher fear, i.e. those who responded ‘agree’ or ‘strongly agree’*.*

### Associations between the cancer fear indicators

Spearman’s correlations showed that cancer as the greatest health fear was significantly correlated with both discomfort thinking about cancer (r = .37, p < .001) and cancer worry (r = .42, p < .001; see Table 2). Chi-square tests showed that of those who had cancer as the greatest health fear, 65% also said that they felt uncomfortable thinking about cancer, compared with 34% of those who did not have cancer as the greatest health fear (*χ*^2^ (1) = 630.8, p < .001). Similarly, 37% of those who had cancer as greatest health fear said they also worried about cancer a lot compared with 8% of those who did not (*χ*^2^ (1) = 696.7, p < .001). These results suggest that having cancer as greatest health fear translates to some extent into high levels of worry and discomfort thinking about the disease.

**Table 2:** **Spearman’s correlations between the three cancer fear indicators, anxiety and general health (N = 6,527)**

	Cancer as greatest health fear	Discomfort thinking about cancer	Cancer worry	General anxiety
Cancer discomfort	.37			
Cancer worry	.42	.35		
General anxiety	.16	.23	.28	
General health	-.07	-.11	-.16	-.29

All presented correlations were significant at p < .001.

### Demographic predictors of cancer fear

Univariate chi-square analyses were used to explore the associations between demographic variables and the three cancer fear indicators. The results are presented in Table 1 and show that significantly more women than men had cancer as greatest health fear (62% vs. 55%), felt uncomfortable thinking about cancer (55% vs. 50%), and worried a lot about cancer (29% vs. 20%). Respondents without educational qualifications (vs. with qualifications) were also more likely to have cancer as greatest health fear (63% vs. 51%), feel uncomfortable thinking about it (56% vs. 46%), and worry a lot about cancer (28% vs. 20%). Respondents from ethnic minority backgrounds were more likely to worry about cancer (37% vs. 24% in the White group), but ethnic differences in discomfort thinking about cancer or having cancer as the greatest health fear were not significant. Age and marital status were not associated with any cancer fear indicator.

We used multiple logistic regression in an analysis that included all demographic characteristics in a single model (Table 3, Model 1). The associations between the demographic variables and the cancer fear indicators were very similar to the results of the univariate analyses, with significant effects of gender and education for all three fear indicators, and of ethnicity for cancer worry. Associations with marital status and age were not significant. All demographic predictors combined explained 2.9% of variance in having cancer as greatest health fear, 1.7% of the variance in discomfort thinking about cancer, and 3.1% of variance in cancer worry.

**Table 3:** **Adjusted Odds Ratios (OR) and 95% Confidence Intervals (CI)**

	Cancer as greatest health fear		Discomfort thinking about cancer		Cancer worry	
	Model 1	Model 2	Model 1	Model 2	Model 1	Model 2
	OR (95% CI)	OR (95% CI)	OR (95% CI)	OR (95% CI)	OR (95% CI)	OR (95% CI)
Gender						
Male	REF	REF	REF	REF	REF	REF
Female	1.34* (1.23-1.50)	1.31* (1.18-1.45)	1.21* (1.09-1.33)	1.15 (1.04-1.27)	1.56* (1.39-1.75)	1.48* (1.33-1.67)
Age						
< 60 years	REF	REF	REF	REF	REF	REF
≥ 60 years	0.92 (0.83-1.01)	0.93 (0.84-1.02)	0.94 (0.85-1.03)	0.95 (0.86-1.05)	0.97 (0.86-1.08)	0.99 (0.88-1.11)
Educational qualifications						
Yes	REF	REF	REF	REF	REF	REF
No	1.70* (1.53-1.88)	1.66* (1.49-1.84)	1.49* (1.35-1.65)	1.43* (1.29-1.58)	1.52* (1.34-1.71)	1.40* (1.24-1.59)
Ethnicity						
White	REF	REF	REF	REF	REF	REF
Not White	1.30 (1.01-1.66)	1.24 (0.96-1.59)	1.35 (1.06-1.72)	1.24 (0.97-1.59)	2.06* (1.60-2.65)	1.85* (1.43-2.39)
Marital status						
Married or cohabiting	REF	REF	REF	REF	REF	REF
Not married or cohabiting	0.95 (0.84-1.06)	0.91 (0.81-1.03)	1.11 (0.99-1.24)	1.05 (0.93-1.17)	1.17 (1.03-1.33)	1.08 (0.95-1.23)
General health						
Excellent/good	-	REF	-	REF	-	REF
Fair/poor		1.05 (0.94-1.18)		1.22* (1.09-1.36)		1.50* (1.33-1.70)
Anxiety						
Low	-	REF	-	REF	-	REF
High		1.50* (1.35-1.66)		1.82* (1.64-2.02)		2.11* (1.90-2.42)
Nagelkerke R^2^	.029	.042	.017	.050	.031	.084
	*χ* ^2^ (5) = 142.6, p < .001	*χ* ^2^ (7) = 206.3, p < .001	*χ* ^2^ (5) = 84.3, p < .001	*χ* ^2^ (7) = 250.9, p < .001	*χ* ^2^ (5) = 138.4, p < .001	*χ* ^2^ (7) = 382.4, p < .001

*p < .001.

*Abbreviations*: *REF* reference category, *OR* odds ratio, *CI* confidence interval.

Adjusted ORs and 95% CIs for the demographic predictors only (Model 1), and the demographic predictors combined with general anxiety and general health (Model 2), for each cancer fear indicator.

### Anxiety and general health as predictors of cancer fear

We then explored whether sociodemographic differences in the three cancer fear indicators were partly driven by differences in general anxiety or health. State anxiety was significantly correlated with all three indicators of cancer fear (r = .16 to .28, all p < .001; see Table 2). Chi-square analyses showed that respondents with high versus low general anxiety were more likely to have cancer as the greatest health fear (65% vs. 54%), feel uncomfortable thinking about cancer (62% vs. 45%) and worry a lot about cancer (34% vs. 17%; see Table 1).

Self-rated health was modestly negatively correlated with the three indicators of cancer fear (r = -.07 to -.16, all p < .001). Respondents with fair/poor versus good/excellent health were more likely to worry about cancer (33% vs. 21%) and feel uncomfortable thinking about cancer (59% vs. 50%), but there were no health differences in having cancer as the greatest health fear.

The second set of regression models (Table 3, Model 2) included the variables in Model 1 plus general health and general anxiety. This made no material difference to the effect sizes associated with gender or SES, and worry about cancer was still higher in ethnic minority respondents. In the fully-adjusted model, general anxiety was an independent predictor of all three fear indicators, while health status was associated with cancer worry and discomfort thinking about cancer. The addition of general health and anxiety to the model increased the proportion of variance explained by the model to 4.2% for having cancer as greatest health fear, 5.0% for discomfort when thinking about cancer, and 8.4% for cancer worry.

## Discussion

More than half this large, community-based sample of 55–64 year-olds in the UK had cancer as greatest health fear and felt uncomfortable thinking about it, and a quarter said they worried ‘a lot’ about cancer. The three indicators were moderately inter-correlated, suggesting some commonality between the three facets of cancer fear. This was supported by finding similar demographic correlates, with all three fear indicators being higher in women and respondents with lower levels of education, and none being associated with age or marital status. Ethnicity was the only demographic variable to show differential associations by fear indicator, with higher worry in non-White groups but no differences in the other indicators. As expected, general anxiety was associated with all three indicators, although the moderate size of the correlations is consistent with cancer fear being distinct from general anxiety. Controlling for general anxiety did not materially change the associations between the sociodemographic predictors and the cancer fear indicators.

The endorsement rate for having cancer as greatest health fear (59%) in this UK sample was similar to previous population surveys conducted in the US, UK, and France, which have found rates of between 35% and 62% [3, 4, 36]. Similar to findings in a French survey [4], more women than men in our study expressed having cancer as greatest health fear. The rate of cancer worry (25%) was also similar to previous studies. General cancer worry was reported in a quarter of UK adults [37], while studies about specific types of cancer showed worry about colorectal cancer in 13% to 23% of community-based samples in the US and UK [34, 38, 39], and worry about lung cancer in about 22% in the US [38]. Worry about breast cancer tends to be higher; around a third of women in the US, UK and Norway reported frequent or considerable breast cancer worry [40–42]. This could be due to the emblematic nature of breast cancer [43], but also to the generally higher rates of cancer worry in women. Similar to US based studies [17, 18], we found that rates of cancer worry tended to be higher in women and people with lower education. Ethnic differences in cancer worry are more difficult to compare across countries, because of the different ethnic minority groups. Overall, comparing our findings with the results of previous studies suggests that gender and education differences in cancer fear may be fairly consistent across Western countries.

The modest inter-correlation between the cancer fear indicators, and the fact that the number of people who identified cancer as their greatest health fear or experienced discomfort thinking about cancer was twice the number of people who experienced cancer worry, suggests that the items used in the current study reflect different aspects of the ‘cancer fear’ construct. This supports suggestions made by other authors that there could be distinct cognitive and affective components of what is often referred to as ‘cancer worry’ [8], and that these components may need to be distinguished to understand the role of cancer fear in cancer-related behaviours [7, 8]. Cancer worry has been associated with higher rates of cancer screening in some studies [7, 44], although this effect has not been entirely consistent (e.g. [20, 21]). But cancer fear may also promote avoidance of the fear stimulus, and has indeed sometimes been mentioned as a barrier to screening [45] and shown to impede cancer screening uptake [39, 42]. The present findings support observations made by other authors that the variation in measurement strategies in studies of cancer fear may have hampered our understanding of its behavioural effects, and that a better understanding of the construct is needed, including an exploration of whether or not it is a multi-dimensional construct [7, 8]. Although the components included in the current study may not be the only relevant ones, our findings give some support to the idea that distinguishing between different components of cancer fear could contribute to understanding of the concept. Further research is needed to determine whether the behavioural effects – for example on screening uptake - also vary by the specific cancer fear component, what additional fear components need to be distinguished, and how all of them could be measured more accurately. Understanding the effects of different fear components may also have implications for the evaluation of public health interventions, which may need to include multiple indicators of fear to accurately assess their effects [for an example of a public health intervention evaluation using multiple fear indicators, see [35]].

The moderate inter-correlations and differential endorsement rates of the three items used in the present study may also suggest a mechanism of protection against worry. High fear states seldom persist unregulated [7], and people who are uncomfortable when thinking about cancer may deploy strategies to reduce their daily worry about cancer. This would be consistent with other common fears, where discussion of the fear object can cause distress but emotional reactions do not necessarily intrude in daily life; as distinct from true cancerophobia [46]. That said, a quarter of the population worrying a lot about cancer, and more than half of the population experiencing discomfort about it might be seen as important issues for quality of life; and public health authorities may be rightly cautious about magnifying cancer fears. However, given that cancer rates are rising, and that there may be a motivating effect of cancer worry on screening uptake, three-quarters of the population *not* worrying about cancer may also be considered a problem. The difficulty of identifying the ‘right’ level of fear for potentially modifiable risks is a general problem in modern societies, and research is needed to get a better understanding of the balance.

The impact of cancer fear on national healthcare systems may be considerable. High cancer worry may motivate more frequent consultations with healthcare professionals to obtain reassurance [47–49]. Alternatively, for individuals who cope using denial or avoidance, discomfort thinking or talking about cancer could lead to delay in help-seeking for potential cancer symptoms [9, 50, 51] and interfere with cancer screening uptake [40, 52, 53]. It could also affect the success of public education on cancer. Miles et al. [10] showed that people with higher levels of cancer fear were more likely to avoid cancer information, including information on the benefits of early detection, thus potentially perpetuating negative beliefs about the scope to reduce cancer risk.

Previous research in an undergraduate sample found moderate correlations between three indicators of cancer fear and dispositional worry, suggesting that cancer fear may be partly due to, but is also distinct from, general anxiety [12]. A study of prostate cancer worry also found only moderate correlations with trait anxiety [15]. The results of the present study are important because they indicate a similar pattern for general cancer fear in a community sample at an age when the threat of cancer is more relevant.

This study has several limitations. First, it was part of larger study that was not designed primarily to investigate cancer fear, and so the selection of predictor variables may not have been optimal. Nonetheless, the large sample size was an advantage. Additional predictors could be considered in future studies, including personal or family history of cancer and perceived personal risk. Participants were aware that it was a survey about cancer and non-responders may have been even more afraid of cancer than responders. A larger proportion of our sample (63%) than the national average of those born between 1936 and 1945 (45%; [54]) reported not having any educational qualifications, although this is unlikely to have influenced the associations with cancer fear that were found in this study. The large proportion of participants without educational qualifications may be due to the location of the General Practices through which they were recruited, which were in more deprived areas of the country. Consistent with the proportion of ethnic minorities in the older British population [55], the majority (96%) of respondents in our sample were from a White background, which limited the power of the study to detect ethnic differences and made it difficult to interpret our findings about the influence of ethnicity on cancer fear. In addition, some evidence suggests that cancer fear is generally lower in those who are older [8], but investigation of age effects was restricted by the narrow age-range of the sample. Lastly, the three components of cancer fear were each measured with single items to reduce participant burden in the main study. There are validity problems associated with single item measures including limited reliability and a limit on the maximum size of any associations, although the effects are offset to some extent by the large sample size.

## Conclusions

Cancer’s highly feared status was endorsed by the majority of a large community sample of 55–64 year-olds, and this was associated both with discomfort in thinking about cancer and frequent worry. Women and people with less education or from ethnic minority backgrounds are disproportionately affected, independent of health status and general anxiety. Because cancer fear is an unpleasant emotion, as well as potentially influencing cancer-protective behaviours, it is important to gain a better understanding of its origins and consequences, and find ways to minimise its impact on quality of life without undermining participation in cancer prevention.

## Abbreviations

FS: Flexible sigmoidoscopy
GP: General practice
HINTS: Health information national trends survey
OR: Odds ratio
SES: Socioeconomic status
SPSS: Statistical package for the social sciences
STAI: (Spielberger’s) State trait anxiety index.
